# Significance of recombinant human growth hormone therapy in promoting growth and development of children with idiopathic short stature

**DOI:** 10.12669/pjms.38.7.6535

**Published:** 2022

**Authors:** Wenbiao Han, Jing Zhang, Tao Song, Yanni Han

**Affiliations:** 1 Wenbiao Han, Department of Endocrinology, Qingyang people’s Hospital, Qingyang 745000, Gansu Province, P.R. China; 2 Jing Zhang, Department of Gynecology and Obstetrics Qingyang people’s Hospital, Qingyang 745000, Gansu Province, P.R. China; 3 Tao Song, Department of Pharmacy, Qingyang people’s Hospital, Qingyang 745000, Gansu Province, P.R. China; 4 Yanni Han, Department of Endocrinology, Qingyang people’s Hospital, Qingyang 745000, Gansu Province, P.R. China

**Keywords:** Idiopathic Short Stature (ISS) in children, Recombinant human growth hormone, Height standard deviation score, Bone age

## Abstract

**Objective:**

To explore the significance of recombinant human growth hormone(rhGH) therapy in promoting the growth and development of children with idiopathic short stature (ISS).

**Methods:**

Medical records of 95 children with ISS, treated in our hospital from July 2019 to July 2020, were retrospectively selected and divided into two groups based on the received treatment. Of them, 41 patients received routine treatment (Group-I) and 54 patients received a combination of routine and rhGH treatment (Group-II). The levels of serum insulin-like growth factor-1 (IGF-1), bone age, growth velocity, height standard deviation score (Ht-SDS), and adverse reactions were compared and analyzed between the two groups.

**Results:**

After treatment, IGF-1, bone age, growth velocity, and Ht-SDS in Group-II were higher than those in Group-I (P < 0.05); After treatment, the incidence of adverse reactions in Group-II was 7.41%, which had no significant difference compared with 7.32% in Group-I (P > 0.05).

**Conclusion:**

In the treatment of children with Idiopathic Short Stature (ISS), the choice of rhGH can further improve the curative effect, promote the growth and development of children, without significant adverse reactions.

## INTRODUCTION

Idiopathic Short Stature(ISS), that is, short stature without underlying pathological conditions and normal growth hormone (GH) levels, is the most common cause of short stature in childhood.^1^,^2^ At present , the pathogenesis of idiopathic short statue has not been clarified. Without targeted intervention, the final height of affected children can be significantly lower than the genetic target height. This may have a profound effect on their physical and mental health, reduce their learning ability and social ability, and increase the risk of self-mutilation and suicide.^3^

In the past, children were mainly offered nutritional support and life interventions that promoted the increase in children’s’ height and weight to a certain extent. However, the overall effect was not ideal, and the rate of growth and development was relatively slow. Recent advances in the clinical research of childhood ISS led to understanding that the GH insulin-like growth factor axis is the most important neuroendocrine axis regulating children’s growth and development.^4^

GH is mainly secreted by the anterior pituitary, a kind of peptide hormone. It has the effect of promoting growth and plays an important role in the process of human development.^5^ Therefore, rhGH gradually became a treatment of choice for childhood ISS. Supplemented exogenous rhGH can fully function in growth and development, promote the division and proliferation of cartilage plate cells, and stimulate child’s height and weight.^6^ The main goal of this retrospective study was to further explore the effect of exogenous rhGH therapy in promoting growth and development of children with Idiopathic Short Stature(ISS).

## METHODS

Medical records of 95 children (48 males and 47 females) with ISS treated in our hospital from July 2019 to July 2020 were retrospectively selected and divided into two groups based on the treatment mode. Of them, 41 children that received routine treatment comprised Group-I. Briefly, children maintained balanced nutrition, moderate exercise, adequate sleep, quantitative calcium and vitamin B_12_ supplement and oral lysine glucozine tablets [taiyangshi (Tangshan) Pharmaceutical Co., Ltd., H20053899,] one tablet/time, one time/day for 12 months. Children (n=54) that received rhGH treatment comprized Group-II. In addition to the routine treatment, children in this Group-were treated with rhGH (Changchun Jinsai Pharmaceutical Co., Ltd., S10980101) 0.15IU/(kg·day). Intramuscular injection was given0.5~1 hour before going to bed around the umbilicus and the middle, front and outer sides of the thigh. Different points were selected for injection each time for 12 months.

### Inclusion criteria:

- In similar living environment, the height is lower than 2 standard deviations or below the 3rd percentile of the average height of normal people of the same age, sex and race (refer to the 2005 Chinese children’s height standard);^7^
- The age of 6 to 12 years;
- The growth rate is slow, but the birth weight and length are within the normal range.
- Peripheral blood GH stimulation peak ≥10μg/L (Using the experimental method of combined excitation of levodopa and insulin)
- Chromosome examination normal
- No relevant treatment received in recent three months before enrollment;
- Complete medical history.

### Exclusion criteria:

- Congenital malformation and chromosome abnormalities;
- Systemic, nutritional and consumptive diseases;
- Severe organic diseases;
- The second sexual sign appeared before the age of eight for girls and nine for boys;
- Puberty began to appear during treatment;
- Bone age closure during treatment;
- Mental and psychological diseases;
- Allergy to the study medications.

This study was approved by the medical ethics association of our college (No. QRYKJ2021-132, Date: 2021-12-28).

Basic information of children and the following indicators were collected before treatment and after 12 months of treatment: 1) Serum insulin-like growth factor-1 (IGF-1) [Blood samples were collected and detected by automatic chemiluminescence immunoanalyzer (IMMULITE 2000) and supporting reagent]. 2) Bone age [Bone age (BA) was measured by X-ray detector (RAYSAFE X2, FLUKE, USA)].3) Growth velocity and height standard deviation value (Ht-SDS) [Ht-SDS= (measured height - average height of children of the same age) / height standard deviation of children of the same age]. 3)Adverse reactions (such as lower limb edema, elevated blood glucose, hypothyroidism, headache).

SPSS22.0 was used to analyze and process the collected data, [n (%)] represented the non-grade count data using *χ^2^* test method; (*X̅*±*S*) was used to represent measurement data. T-test was performed in cases of normal distribution, with the test level of *a*=0.05. Rank sum test was done in cases of not normal distribution. When *P*< 0.05, the difference was considered statistically significant.

## RESULTS

A total of 95 children met the inclusion criteria, including 48 males and 47 females. The age of children ranged from 6 to 12 years, with an average of (10.01±1.71) years. The height was 76~145cm, with an average of (122.38±16.44) cm. Of them, 41 patients received routine treatment (Group-I) and 54 received routine treatment in combination with rhGH therapy (Group-II).

There was no significant difference in basic clinical characteristics between the two groups (*P*>0.05) (Table-I). There was no significant difference in IGF-1, bone age, growth velocity and Ht-SDS between the two groups before treatment ((*P*>0.05)), but they were significantly improved after 12 months of treatment (*P*<0.05), and the degree of improvement in Group-II was significantly higher than that in Group-I (*P*<0.05) (Table-II and Table-III). After 12 months of treatment, the incidence of adverse reactions in Group-II was 7.41%, which had no significant difference compared with 7.32% in Group-I (*P*>0.05) (Table-IV).

**Table-I:** Comparison of general information between the two groups [n (%),X̅±S ].

Group	n	Gender (Male/Female)	Age (Year)	Height (cm)
Group-I	41	20/21	10.02±1.81	123.29±18.60
Group-II	54	28/26	10.17±1.64	121.68±14.73
χ^2^/t	-	0.088	0.400	0.470
P	-	0.767	0.690	0.639

**Table-II:** Comparison of IGF-1 level and bone age between the two groups before and after treatment (*X̅*±*S*).

Group(n)	IGF-1(ng/L)		t	P	Bone age(year)		t	P
	
	Before treatment	12 months after surgery			Before treatment	12 months after surgery		
Group-I(n=41)	112.24±32.52	207.51±38.17	102.889	<0.001	8.14±1.78	9.34±1.84	19.073	<0.001
Group-II(n=54)	109.83±35.68	291.42±36.14	231.236	<0.001	7.94±1.62	10.18±1.60	26.982	<0.001
t	0.339	10.940	-	-	0.576	2.384	-	-
P	0.736	<0.001	-	-	0.566	0.019	-	-

**Table-III:** Comparison of growth rate and standard deviation of height between two groups (*X̅*±*S*).

Group(n)	Growth velocity(cm/year)		t	P	Ht-SDS		t	P
	
	Before treatment	12 months after surgery			Before treatment	12 months after surgery		
Group-I(n=41)	4.44±1.03	6.09±1.20	8.203	<0.001	-2.22±0.34	-1.70±0.29	22.237	<0.001
Group-II(n=54)	4.25±0.94	8.53±1.08	23.411	<0.001	-2.31±0.30	-1.49±0.23	37.259	<0.001
t	0.953	10.351	-	-	1.283	0.679	-	-
P	0.343	<0.001	-	-	0.203	<0.001	-	-

**Table-IV:** Comparison of the occurrence of adverse reactions between the two groups [n (%)].

Group	n	Adverse reactions				Total

		Lower extremity edema	Elevated blood sugar	Hypothyroidism	Headache	
Group-I	41	1(2.44)	1(2.44)	0(0.00)	1(2.44)	3(7.32)
Group-II	54	1(1.85)	1(1.85)	1(1.85)	1(1.85)	4(7.41)
χ^2^	-	-	-	-	-	0.003
P	-	-	-	-	-	0.987

## DISCUSSION

Idiopathic Short Stature(ISS) is a common type of children’s dwarfism. The etiology is complex, and its pathogenesis is not clear at present. According to Jawa A *et al*^8^ whether children with ISS treated with GH experience the same metabolic benefits as children with GH deficiency (GHD) treated with GH, the results showed that children with ISS also benefit from GH treatment, with increased height and no serious negative metabolic results. If it is not intervened in time, the height of children cannot be, and the height of their successful years is significantly lower than that of their peers, resulting in a serious decline in their quality of life.^9^ In 2003, the U.S. Food and Drug Administration approved rhGH in the treatment of ISS. Since then, a large number of clinical studies have begun to treat ISS through this program. The results show that it can promote the growth rate of children.^10^

In this study, rhGH was used to treat children with ISS, the results showed that the total effective rate of Group-II was 94.44%, higher than 75.61% of Group-I (*P*<0.05), suggesting that the implementation of rhGH treatment in children with ISS is helpful to further improve the curative effect. Soliman A *et al*^11^ studied the effect of growth hormone on children with ISS The results showed that IGF-1, BMI, htsds and bone growth rate were significantly higher than those of untreated ISS patients one year after treatment, which is consistent with our results. In addition, Paltoglou G *et al*^12^ conducted a systematic literature search and finally included 21 studies for meta-analysis, the results showed that the height and growth of children receiving rhGH treatment at the end of the first year was significantly higher than that of the control group, this effect lasted until the second year of treatment, and the difference between the two groups was equal to 5.3 cm (95% CI: 3.4-7 cm) for male and 4.7 cm (95% CI: 3.1-6.3 cm) for female patients. In the whole process of growth and development, rhGH plays an important role in protein synthesis and growth, lipolysis, trace element absorption and so on.^13^ rhGH is similar to human endogenous growth hormone, after subcutaneous injection, it can supplement exogenous growth hormone, and be fully functional and effective in promoting growth and development.

In the treatment of childhood Idiopathic Short Stature(ISS) , BMI, BA and Growth Hormone GV are common efficacy evaluation indicators, which can directly reflect the growth and development of children after treatment.^14^ In this study, BMI, BA and GV in Group-II were better than those in Group-I (*P*<0.05), suggesting that rhGH treatment for childhood ISS is helpful to further promote the growth and development of children. Gohil A *et al*^15^ gave rhGH treatment to children with ISS, similarly, to the results of our study, they showed that growth and development of children significantly improved after the treatment. The application of rhGH treatment can meet the GH needs of children in the process of growth and development, promote bone absorption, bone formation and bone accumulation, improve bone growth and development, and improve GV and BA levels.^16^ In addition, due to adequate rhGH supplementation, children’s height shows a catch-up growth trend, while their weight generally does not increase significantly. Therefore, it can promote the improvement of BMI value, leading to the healthy growth and development. Additionally, Wu B *et al*^17^ study showed that the rhGH can act on the growth hormone insulin-like growth factor axis, interacting with the growth hormone receptor on the target organ, and with IGF-1, IGFBP-3 and acid unstable units to form a ternary complex. It can promote bone growth, cell division and proliferation.

Recent studies have shown that long-term application of rhGH has the risk of rising blood glucose and hypothyroidism, but other studies have shown that the effect of rhGH on blood glucose and thyroid function is not obvious.^18^ In this study, the incidence of adverse reactions was comparable in both groups (*P*>0.05), suggesting that rhGH treatment for childhood ISS will not increase the incidence of adverse reactions. Our results are in agreement with the study of Heidvall K *et al*^19^ that compared and analyzed the adverse reactions of routine treatment and rhGH treatment in children with ISS, and found that there was no significant difference in adverse reactions between the two treatments. At present, there is no clear standard for the dosage of rhGH in the treatment of childhood ISS. Some guidelines recommend the dosage of 0.15~0.2IU/(kg·d), while some clinically recommend the dosage of 0.1~0.2IU/(kg·d). In this study, the dosage was 0.15IU/(kg·d), which is considered moderate was used. Our study shows that it can not only meet the needs of rhGH in the process of children’s growth and development, but also has ideal safety.

### Limitations

There are many limitations in this study. For example, only 95 patients received in our hospital in recent one year were selected, and there are few observation indicators. Moreover, short-term follow-up observation up to 12 months after treatment has high subjectivity, which may make the conclusion one-sided and limited.

## CONCLUSION

In the treatment of children with Idiopathic Short Stature(ISS), the choice of rhGH can further improve the curative effect, promote the growth and development of children, without significant adverse reactions.

### Authors’ Contributions:

**WH** conceived and designed the study.

**JZ, TS and YH** collected the data and performed the analysis.

**WH and JZ** was involved in the writing of the manuscript and is responsible for integrity of the study.

All authors have read and approved the final manuscript.
